# Dietary Antioxidant Intake and Sleep Quality: Combined Effects on Chronic Obstructive Pulmonary Disease in NHANES 2005–2008 and Mendelian Randomization Analysis

**DOI:** 10.1002/fsn3.71209

**Published:** 2025-11-17

**Authors:** Kaiyue Wang, Xinjing Lou, Shenghan Wang, Qunhao Huang, Lingna Liu, Jiangnan Lin, Linyu Wu, Chen Gao

**Affiliations:** ^1^ Department of Radiology The First Affiliated Hospital of Zhejiang Chinese Medical University (Zhejiang Provincial Hospital of Chinese Medicine) Hangzhou China; ^2^ The First School of Clinical Medicine Zhejiang Chinese Medical University Hangzhou China

**Keywords:** COPD, DAQS, Mendelian randomization, NHANES, PSQI

## Abstract

Both dietary antioxidant quality score (DAQS) and sleep quality are associated with chronic obstructive pulmonary disease (COPD), but their joint influence remains unclear. This investigation examined the associations among DAQS, sleep quality, and COPD in the US adult population utilizing data from the National Health and Nutrition Examination Survey (NHANES). Data from 8752 individuals participating in the NHANES 2005–2008 cycles were analyzed. The associations between COPD risk and both individual and joint effects of DAQS and Pittsburgh Sleep Quality Index (PSQI) were examined using multivariate logistic regression and subgroup analyses. Additionally, potential causal links were explored through two‐sample and multivariable Mendelian randomization (MR) analysis. After adjusting for confounders, higher DAQS was negatively associated with COPD (OR = 0.92, 95% CI: 0.85–0.99, *p* = 0.029), while PSQI was positively related (OR = 1.07, 95% CI: 1.05–1.09, *p* < 0.001). Relative to individuals with low DAQS and poor sleep quality, the OR for COPD was 0.68 (95% CI: 0.48–0.98) in those with high DAQS and poor sleep quality; 0.54 (95% CI: 0.37–0.79) with low DAQS and good sleep quality; and 0.45 (95% CI: 0.29–0.69) with high DAQS and good sleep quality. Mendelian randomization analysis suggested causal effects of variation in diet and sleep duration on COPD. Based on analyses of a large, nationally representative dataset and Mendelian Randomization studies, our research demonstrated significant associations between DAQS, sleep quality, and COPD. This study is the first to establish the synergistic effect of high DAQS and good sleep quality in mitigating the risk of developing COPD.

## Graphical abstract

Analysis of NHANES data revealed that a higher dietary antioxidant quality score (DAQS) was associated with lower COPD risk, while poor sleep quality (PSQI) was associated with higher risk. The combination of high DAQS and good sleep quality was associated with the greatest risk reduction (OR = 0.45). Mendelian randomization analysis supported potential causal roles.

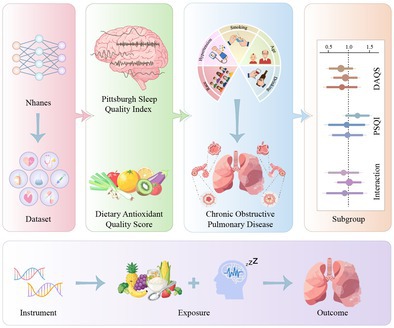

## 1 Background

Chronic obstructive pulmonary disease (COPD) is a widespread respiratory disorder marked by irreversible airflow obstruction and progressive loss of lung function (GBD 2015 Chronic Respiratory Disease Collaborators 2017). Worldwide, COPD is the fourth major cause of mortality, with annual deaths reaching 3.5 million in 2021, accounting for about 5% of all deaths worldwide (World Health Organization 2024, https://www.who.int/news‐room/fact‐sheets/detail/chronic‐obstructive‐pulmonary‐disease‐(copd)). This condition imposes significant socioeconomic and healthcare resource burdens worldwide, emphasizing the urgent need for effective preventive and therapeutic strategies (Christenson et al. 2022).

The dietary antioxidant quality score (DAQS) is an integrated measure of dietary antioxidant consumption, comprising vitamins A, C, and E, as well as zinc, selenium, and magnesium (US Department of Health and Human Services and US Department of Agriculture 2015, https://odphp.health.gov/our‐work/food‐nutrition/previous‐dietary‐guidelines/2015). This metric is calculated by comparing individual antioxidant consumption levels against their corresponding recommended daily intake (RDI) (Ahmadi Vasmehjani et al. 2024; Tur et al. 2005). Previous research has shown that DAQS has important implications for hypertension, diabetes, and childhood asthma (Wang et al. 2024; Lin et al. 2024). Accumulating evidence suggests that dietary antioxidants function as free radical scavengers and suppress reactive oxygen species production, thereby conferring significant protective functions in diverse metabolic and redox pathways (Shahavandi et al. 2021; Stańczyk et al. 2005; Willcox et al. 2004). Patients with COPD often experience elevated ROS levels and heightened oxidative stress, especially during acute exacerbations (Barnes 2020; Schaberg et al. 1995). Earlier studies have found that inadequate intake of individual antioxidants, including vitamins A, C, and E, correlates with COPD risk; however, the relationship between DAQS and COPD remains unclear (Lei et al. 2022; Peh et al. 2017). Elucidating this association is vital for developing targeted interventions aimed at preventing or slowing the progression of COPD.

The Pittsburgh Sleep Quality Index (PSQI) is extensively employed in both clinical practice and research to evaluate sleep quality (Buysse et al. 1989; Mollayeva et al. 2016). Poor sleep quality is a key element in COPD (Agusti et al. 2011), given its frequent association with nocturnal hypoxemia, nighttime dyspnea, nocturnal coughing, and comorbid conditions that may heighten the likelihood of developing COPD (Agusti et al. 2011; Weitzenblum and Chaouat 2004).

Growing evidence suggests associations between COPD and both low dietary antioxidant intake and poor sleep quality (Zheng et al. 2024; Lin et al. 2023), and that these two unfavorable conditions often occur together (Vaccaro et al. 2020). However, how dietary antioxidant intake and PSQI jointly affect COPD remains unclear. Therefore, this study analyzed data from the National Health and Nutrition Examination Survey (NHANES) to investigate the relationships between the DAQS, PSQI, and COPD, as well as their combined effects on COPD in the US adult population.

## 2 Methods

### 2.1 Study Design and Participants

**FIGURE 1:**
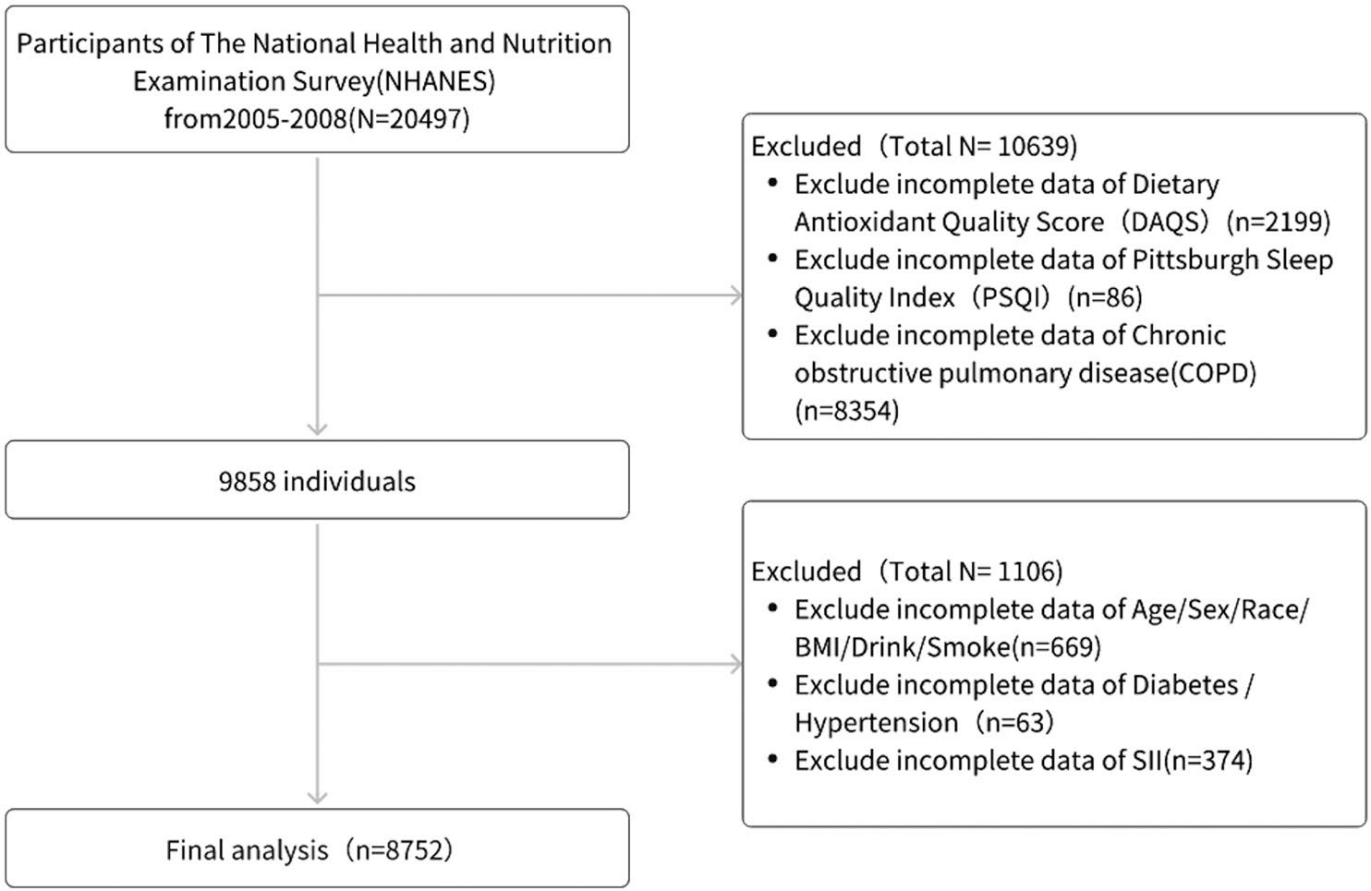
Protocol for selection of study population.

The NHANES evaluates the health and nutritional status of the US population using complex, multistage probability sampling procedures. Each survey cycle collects demographic data, diet, physical examinations, laboratory tests, and questionnaires. The study protocols were approved by the NCHS Ethics Review Board, with participants providing written consent prior to enrollment. This analysis utilized data from two consecutive NHANES cycles (2005–2006 and 2007–2008), which included 20,497 eligible participants. After excluding individuals with missing data, 8752 remained for the final analysis (Figure 1).

### 2.2 Calculation of the DAQS

The DAQS was determined based on six essential antioxidants: vitamins A, C, and E, along with selenium, magnesium, and zinc. Each micronutrient's daily intake was evaluated against its RDI as outlined in the Dietary Guidelines for Americans 2015–2020 (US Department of Health and Human Services and US Department of Agriculture 2015, https://odphp.health.gov/our‐work/food‐nutrition/previous‐dietary‐guidelines/2015). A score of 0 was assigned for intake below two‐thirds of the RDI, while 1 point was allocated for intake at or above this threshold. The total DAQS was obtained by summing these individual scores, producing a scale of 0 to 6, where higher values reflect increased antioxidant intake. DAQS values were subsequently categorized into low quality (1–4) and high quality (5–6) (Shi and Fang 2024).

### 2.3 Assessment of Sleep Quality

Sleep quality and patterns were evaluated using the PSQI, a widely adopted tool for both adolescents and adults (Buysse et al. 1989; Vézina‐Im et al. 2018). In the 2005–2008 NHANES dataset, six self‐reported items contributed to PSQI scores in three domains: sleep latency, sleep disturbances, and daytime dysfunction. The sum of these domain scores yielded a total PSQI value of 0–23, with higher values indicating decreased sleep quality (Wang et al. 2022). A cutoff value above 5 was used to classify participants as having poor sleep quality (De Moura et al. 2025).

### 2.4 Assessment of COPD

COPD status was ascertained through self‐reported physician diagnoses. Participants were determined to have COPD based on positive responses to either of the following: “Has a doctor or other health professional ever told you that you have chronic bronchitis?” or “Has a doctor or other health professional ever told you that you have emphysema?” Participants responding “no” to both questions were designated as controls.

### 2.5 Assessment of Covariates

Covariates associated with COPD included demographic factors (age, sex, race), clinical parameters (body mass index, hypertension, diabetes), lifestyle factors (smoking status, alcohol consumption), and the systemic immune‐inflammation index (SII). Age was stratified into three groups: ≤ 45, 46–60, and > 60 years. Sex categories included male and female, and racial groups comprised Mexican American, non‐Hispanic Black, non‐Hispanic White, other Hispanic, and other races. BMI calculation used weight (kg) divided by height (m) squared. Smokers were defined by lifetime cigarette consumption (≥ 100), while drinkers were identified by annual alcohol intake (≥ 12 drinks). Hypertension criteria included blood pressure readings (systolic ≥ 130 mmHg or diastolic ≥ 80 mmHg) or physician diagnosis. Diabetes was confirmed by physician diagnosis, clinical parameters (HbA1c ≥ 6.5% or fasting glucose ≥ 7.0 mmol/L), or insulin use. SII derivation involved the ratio of platelet–neutrophil product to lymphocyte count.

### 2.6 Statistical Analysis

Survey‐weighted statistical procedures addressed the intricate sampling design, including stratification and clustering effects. Consistent with NHANES guidelines, the sampling weights corresponding to the smallest subsample were used in the analysis. Data distribution guided the presentation format: mean ± standard deviation (SD) for normal distributions, and medians with interquartile ranges for non‐normal distributions. Categorical variables were expressed as percentages. Statistical differences among COPD and non‐COPD groups underwent analysis through parametric or non‐parametric tests (*t*‐test or Kruskal–Wallis) for continuous variables, with categorical variables assessed via Chi‐squared test. Logistic regression evaluated associations of DAQS and PSQI with COPD using three models: unadjusted (Model 1), basic demographic adjustment (Model 2: age, sex, race), and full covariate adjustment (Model 3: added BMI, smoking status, alcohol consumption, hypertension, diabetes, and SII). Interaction terms were introduced to determine whether the joint effects of DAQS and PSQI exceeded their combined individual effects on both additive and multiplicative scales. On the additive scale, the relative excess risk due to interaction (RERI) was calculated, adhering to the method proposed by Knol and VanderWeele (2012). On the multiplicative scale, the ratio of odds ratios (ROR) was used to gauge potential interactions. Possible heterogeneity and interaction effects were explored through stratified analyses across different groups. Statistical analyses employed R 4.3.3, considering *p* < 0.05 (two‐sided) as statistically significant.

### 2.7 Mendelian Randomization Analysis

Two‐sample Mendelian randomization (MR) and multivariable MR (MVMR) analysis was performed to investigate the causal relationships among genetically predicted dietary quality, sleep quality, and COPD. Genome‐wide association study (GWAS) data for sleep quality and COPD were obtained from the UK Biobank (Bycroft et al. 2018), and data on dietary quality were retrieved from the European Bioinformatics Institute. No sample overlap was identified because the populations included in these GWAS datasets were drawn from different sources.

To establish causality, the MR analysis was designed to meet three fundamental assumptions: (1) each genetic variant must be associated with either dietary or sleep quality; (2) it must not be associated with confounders; and (3) it must affect COPD only through its influence on dietary or sleep quality. Variants meeting these criteria were selected as follows (Burgess et al. 2017). Two‐sample MR analysis and multivariate MR (MVMR) selected variants demonstrated genome‐wide associations (*p* < 1 × 10^−5^) and underwent linkage disequilibrium pruning (*r* ^2^ < 0.001 within a 10,000 kb window). The main analysis employed inverse variance weighted (IVW), supported by MR‐Egger regression and weighted median (WM) approaches. Odds ratios (ORs) and 95% confidence intervals (CIs) were standardized to reflect changes per SD increase in exposure variables.

Assessment of heterogeneity and pleiotropy employed Cochran's Q test, MR‐Egger intercept analysis, and MR‐PRESSO global test to ensure robust results, along with calculation of the F‐statistic for genetic instrumental variables (IVs) to assess instrument strength.

## 3 Result

### 3.1 Baseline Data Characteristics

This study examined 8752 participants (weighted population: 210,293,024; weighted proportion of females: 52.36%). Compared to the non‐COPD group, participants with COPD were older, had higher BMI and SII values, and included larger proportions of females, smokers, and non‐Hispanic White individuals. The COPD group also demonstrated a higher prevalence of hypertension and diabetes, higher mean PSQI scores (*p* < 0.001), and a statistically significant difference in DAQS (*p* = 0.004) (Table 1).

**TABLE 1:** Baseline characteristics according to COPD status.

Characteristic	Overall (*n* = 8752)	Non‐COPD (*n* = 8068)	COPD (*n* = 684)	*p*
Age, years, *n* (%)				< 0.001
≤ 45	3939 (50.21)	3762 (51.74)	177 (32.13)	
46–60	2066 (27.68)	1888 (27.48)	178 (30.02)	
> 60	2747 (22.12)	2418 (20.78)	329 (37.86)	
Sex, *n* (%)				0.006
Female	4479 (52.36)	4093 (51.70)	386 (60.22)	
Male	4273 (47.64)	3975 (48.30)	298 (39.78)	
Race, *n* (%)				< 0.001
Mexican American	1641 (8.47)	1595 (8.93)	46 (2.96)	
Non‐Hispanic black people	1755 (10.54)	1644 (10.73)	111 (8.33)	
Non‐Hispanic white people	4383 (72.24)	3931 (71.60)	452 (79.68)	
Other Hispanic	652 (3.87)	607 (3.94)	45 (3.01)	
Other races	321 (4.89)	291 (4.79)	30 (6.02)	
BMI, kg/m^2^, Median (*P*_25_, *P*_75_)	27.49 (24.05, 31.77)	27.38 (24.02, 31.60)	28.74 (24.49, 33.64)	0.003
Smoke, *n* (%)				< 0.001
Yes	4197 (48.54)	3708 (46.70)	489 (70.15)	
No	4555 (51.46)	4360 (53.30)	195 (29.85)	
Drink, *n* (%)				0.600
Yes	6130 (75.46)	5653 (75.52)	477 (74.75)	
No	2622 (24.54)	2415 (24.48)	207 (25.25)	
Hypertension, *n* (%)				0.037
Yes	1033 (9.79)	933 (9.51)	100 (13.08)	
No	7719 (90.21)	7135 (90.49)	584 (86.92)	
Diabetes, *n* (%)				< 0.001
Yes	1308 (10.53)	1155 (10.01)	153 (16.71)	
No	7444 (89.47)	6913 (89.99)	531 (83.29)	
SII, 1000 cell/μL, Median (*P*_25_, *P*_75_)	527.23 (384.48, 735.78)	522.00 (380.63, 728.31)	592.97 (423.53, 793.33)	< 0.001
DAQS, Median (*P*_25_, *P*_75_)	4.00 (3.00, 5.00)	4.00 (3.00, 5.00)	4.00 (2.00, 5.00)	0.004
PSQI, Median (*P*_25_, *P*_75_)	7.00 (4.00, 11.00)	7.00 (4.00, 11.00)	9.00 (5.00, 14.00)	< 0.001

Abbreviations: COPD, chronic obstructive pulmonary disease; DAQS, dietary antioxidant quality score; PSQI, Pittsburgh Sleep Quality Index; SII, systemic immune‐inflammation index.

### 3.2 Associations of DAQS and PSQI With COPD

The associations of DAQS and PSQI with COPD risk were examined using three logistic regression models (Tables 2 and 3). In the final model, DAQS was negatively associated with COPD (OR = 0.92, 95% CI: 0.85–0.99, *p* = 0.029), while PSQI was positively associated with COPD (OR = 1.07, 95% CI: 1.05–1.09, *p* < 0.001). Participants with high dietary quality (DAQS = 5–6) demonstrated a 28% lower risk of COPD versus those with low dietary quality (DAQS = 1–4). Conversely, those reporting poor sleep quality experienced a 75% higher COPD risk than those with good sleep quality.

**TABLE 2:** Association between DAQS and COPD.

Participants	Model 1		Model 2		Model 3	
	OR (95% CI)	*p*	OR (95% CI)	*p*	OR (95% CI)	*p*
DAQS	0.90 (0.83–0.96)	0.004	0.88 (0.82–0.95)	0.001	0.92 (0.85–0.99)	0.029
DAQS = 1–4	1.0 [Reference]		1.0 [Reference]		1.0 [Reference]	
DAQS = 5–6	0.70 (0.56–0.88)	0.003	0.65 (0.52–0.82)	< 0.001	0.72 (0.56–0.92)	0.011

*Note:* Model 1 without adjustments; Model 2 additionally adjusted for age, sex, race; Model 3 additionally adjusted for BMI, smoking, drinking, hypertension, diabetes, and SII.

Abbreviations: CI, confidence interval; DAQS, dietary antioxidant quality score; OR, odds ratio.

**TABLE 3:** Association between PSQI and COPD.

Participants	Model 1		Model 2		Model 3	
	OR (95% CI)	*p*	OR (95% CI)	*p*	OR (95% CI)	*p*
Sleep quality	1.08 (1.06–1.10)	< 0.001	1.08 (1.06–1.11)	< 0.001	1.07 (1.05–1.09)	< 0.001
Good sleep quality	1.0 [Reference]		1.0 [Reference]		1.0 [Reference]	
Poor sleep quality	1.81 (1.43–2.29)	< 0.001	1.81 (1.43–2.29)	< 0.001	1.75 (1.37–2.23)	< 0.001

*Note:* Model 1 without adjustments; Model 2 additionally adjusted for age, sex, race; Model 3 additionally adjusted for BMI, smoking, drinking, hypertension, diabetes, and SII.

Abbreviations: CI, confidence interval; OR, odds ratio; PSQI, Pittsburgh Sleep Quality Index.

### 3.3 Combined Effects of DAQS and PSQI on COPD Incidence

Analysis involved categorizing participants into four groups according to dietary quality (low vs. high) and sleep quality (poor vs. good). Using participants with low dietary quality and poor sleep as a reference, individuals with high dietary quality and poor sleep (OR = 0.68, 95% CI: 0.48–0.98), those with low dietary quality and good sleep (OR = 0.54, 95% CI: 0.37–0.79), and participants with both high dietary quality and good sleep (OR = 0.45, 95% CI: 0.29–0.69) showed a decreased risk (Table 4).

**TABLE 4:** Associations between DAQS and PSQI with COPD in NHANES.

Participants	Model 1		Model 2		Model 3	
	OR (95% CI)	*p*	OR (95% CI)	*p*	OR (95% CI)	*p*
Subgroup 1	1.0 [Reference]		1.0 [Reference]		1.0 [Reference]	
Subgroup 2	0.65 (0.47–0.90)	0.011	0.61 (0.43–0.86)	0.008	0.68 (0.48–0.98)	0.039
Subgroup 3	0.51 (0.35–0.73)	< 0.001	0.51 (0.35–0.75)	0.001	0.54 (0.37–0.79)	0.003
Subgroup 4	0.43 (0.28–0.65)	< 0.001	0.40 (0.27–0.60)	< 0.001	0.45 (0.29–0.69)	0.001

*Note:* Subgroup 1 is DAQS = 1–4 and poor sleep quality; subgroup 2 is DAQS = 5–6 and poor sleep quality; subgroup 3 is DAQS = 1–4 and good sleep quality; subgroup 4 is DAQS = 5–6 and good sleep quality; Model 1 without adjustments; Model 2 additionally adjusted for age, sex, race; Model 3 additionally adjusted for BMI, Smoke, drink, hypertension, diabetes, and systemic immune‐inflammation index.

Abbreviations: CI, confidence interval; OR, odds ratio.

### 3.4 Interaction Test

Significant interactions were observed on both additive and multiplicative scales. In the final model, the RERI was 0.23, indicating that dietary antioxidant intake and sleep quality demonstrated synergistic effects beyond their impacts on COPD. The ratio of odds ratios (ROR) of 1.23 demonstrated a greater than multiplicative interaction between these factors. In addition, the ROR in the final model was 1.23, indicating an interaction effect that surpassed the multiplicative combination of their independent contributions.

### 3.5 Subgroup Analysis

Subgroup analyses evaluated whether the associations of DAQS and PSQI with COPD, as well as their joint effects, differed by sex, age, race, smoking status, alcohol consumption, hypertension, and diabetes. A significant interaction was observed between race and the association of DAQS with COPD (*p* for interaction = 0.028) (Figure S1). However, no significant interactions were found in the stratified analyses of PSQI with COPD or in the combined effects of DAQS and PSQI on COPD across other variables (all *P* for interactions > 0.05) (Figures S2 and S3). Notably, significant interactions on both additive and multiplicative scales were identified for the combined effects of DAQS and PSQI on COPD among subgroups of males, females, individuals aged 45–60 years, those older than 60 years, other Hispanic populations, smokers, alcohol consumers, and those without diabetes (Table S1).

### 3.6 MR of DAQS and PSQI With COPD

**FIGURE 2:**
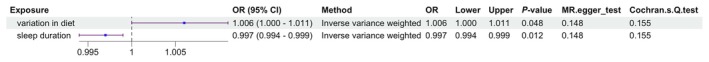
Diet and sleep—COPD multivariate MR analysis and sensitivity analysis. Forest plot showing results from the multivariable Mendelian randomization study assessing the joint causal effects of variation in diet and sleep duration on chronic obstructive pulmonary disease (COPD). CI, confidence interval; OR, odds ratio.

Two‐sample Mendelian randomization (MR) analyses and multivariable MR (MVMR) analyses were employed to investigate the potential causal effects of variation in diet and sleep duration on COPD risk and the joint causal impacts of variation in diet and sleep duration on COPD pathogenesis. In the Two‐sample MR Analysis, the IVW analysis revealed a significant causal effect of genetically predicted variation in diet on COPD risk (OR = 1.010, *p* < 0.001), and a significant causal effect of genetically predicted sleep duration (OR = 0.996, *p* = 0.002) (Figure S4). In multivariable MR analysis employing inverse‐variance weighted (IVW) method, genetically instrumented variation in diet (using genetic instruments for diet) and sleep duration remained associated with COPD risk (OR = 1.006, *p* = 0.048 and OR = 0.997, *p* = 0.012, respectively). These results indicated that there was a joint causal effect of diet change and sleep quality on the risk of COPD (Figure 2). After excluding outliers, MR‐PRESSO analyses produced similar findings, whereas no significant associations emerged in the WM or MR‐Egger models (all *p* > 0.05). Results from Cochran's Q test and the MR‐Egger intercept showed *p* values above 0.05, indicating the absence of heterogeneity and horizontal pleiotropy in these analyses. In the two‐sample MR analyses, all SNPs for both sleep duration and variation in diet yielded F‐statistics above the standard threshold (*F* > 10) (Tables S2 and S3). In the MVMR analyses, variation in diet showed reasonable instrument strength (*F* > 10), whereas the instrument strength for sleep duration was low (*F* = 8.372) (Table S4).

## 4 Discussion

Little research has specifically explored the combined effect of DAQS and PSQI on COPD among US adults. Our study revealed that both low dietary antioxidant intake and poor sleep quality were associated with elevated COPD risk, with their combination conferring a higher risk than either factor alone. Moreover, two‐sample MR results suggested that variation in diet and sleep duration may heighten COPD risk, while the multivariable MR results suggested that there might be a joint causal effect of diet and sleep on COPD.

Previous research demonstrated significant links between inadequate intake of specific dietary antioxidants and COPD (Laudisio et al. 2016; Rodríguez‐Rodríguez et al. 2016; Tabak et al. 1999). For example, measuring vitamins A and C may help identify populations at elevated risk for COPD (Salo et al. 2022). Vitamin E can protect against COPD by downregulating the EGFR/MAPK pathway, thus inhibiting COX2 expression and preventing the nuclear translocation of phosphorylated STAT3 (Zhao et al. 2022). Another study suggested that vitamin C may serve as a supplement to prevent respiratory diseases, including COPD (Ghalibaf et al. 2023). Recent evidence emphasizes that healthy dietary patterns are shaped by both individual nutrients and their synergistic interactions (Morze et al. 2020). Accordingly, assessing overall dietary antioxidant intake may be more meaningful than focusing solely on individual antioxidants (Guasch‐Ferré and Willett 2021; Tapsell et al. 2016). Despite the scarcity of evidence linking overall dietary antioxidant intake to COPD, our study found that participants with higher overall dietary antioxidant intake demonstrated a 28% reduced risk for COPD relative to those with lower intake. This underscores the role of total dietary antioxidant consumption, rather than individual antioxidants alone, in reducing COPD incidence. Promoting diets rich in antioxidant‐containing foods may thus be an effective approach for mitigating COPD risk.

Previous research has indicated that poor sleep quality may exacerbate systemic inflammation through immune dysregulation (Ren et al. 2025), which could indirectly increase the risk of developing COPD. Furthermore, sleep is associated with a loss of accessory respiratory muscle function and supine posture‐induced ventilation‐perfusion mismatch and airflow obstruction (Gao and Zhu 2025), which can worsen lung hyperinflation. These factors collectively increase respiratory load and may promote or exacerbate upper airway obstruction, potentially contributing to COPD incidence. Although no studies have definitively proven that sleep disorders directly increase COPD incidence, our results newly indicate poor sleep quality as a potential risk factor for COPD. Participants reporting poor sleep quality showed a 75% increased risk of COPD versus those who slept well. Additional research is needed to clarify the biological and physiological mechanisms connecting sleep disorders to respiratory health, particularly the association between sleep disorders and new‐onset COPD.

To our knowledge, this study is the first investigation using nationally representative data to examine how dietary antioxidant intake and sleep quality jointly influence COPD. Earlier research has established independent links between sleep disorders, inadequate dietary antioxidant intake, and COPD, but relatively few studies have addressed their combined effects (McNicholas et al. 2013; Biswas et al. 2013; Rahman and Kilty 2006; MacNee 2000). Our results reveal a significant interaction between these two factors on COPD risk, with the magnitude of this interaction reaching statistical significance on both additive and multiplicative scales. These findings suggest that individuals who consume low levels of dietary antioxidants and have poor sleep quality face a substantially higher risk of developing COPD. By examining both factors together, we gained a deeper understanding of their individual and synergistic roles in COPD development, offering important insights for clinical practice. Specifically, our data indicate that participants with both low dietary antioxidant intake and poor sleep quality are at significantly higher risk for COPD than those with only one of these risk factors. Future studies should focus on elucidating the mechanisms through which dietary antioxidant intake and sleep quality affect COPD pathogenesis. Such information could inform health behavior interventions targeting both diet and sleep to help reduce COPD risk.

Subgroup analyses revealed that the protective effect of DAQS against COPD differed significantly across racial groups, with a particularly pronounced effect observed in Non‐Hispanic Whites. Our findings indicate that after adjusting for confounders such as age and sex, each unit increase in dietary antioxidant quality among Non‐Hispanic Whites was associated with an 11% reduction in COPD risk. However, this protective effect was not statistically significant in other racial groups. Notably, recent analysis from the same dataset demonstrated that Non‐Hispanic Whites consistently had higher average dietary vitamin A intake compared to other racial groups (Cheng et al. 2023). Given that vitamin A is a key component of the DAQS, this disparity may partly explain the racial specificity in the protective effect of DAQS (US Department of Health and Human Services and US Department of Agriculture 2015, https://odphp.health.gov/our‐work/food‐nutrition/previous‐dietary‐guidelines/2015). These differences may stem from genetic variations, dietary culture, or socioeconomic factors. Further studies with larger sample sizes and more centers are warranted to validate the specific mechanisms of vitamin A and other antioxidants in the relationship between DAQS and COPD.

Several plausible biological mechanisms may explain the interrelationships between sleep quality, dietary antioxidant intake, and COPD development. Dietary antioxidants such as resveratrol appear to exert antioxidative and anti‐inflammatory effects on COPD cells in vitro and reduce neutrophilic infiltration in the lungs of lipopolysaccharide‐induced rats in vivo (Culpitt et al. 2003; Birrell et al. 2005). Resveratrol has also been shown to slow accelerated lung aging in a mouse model lacking telomerase (Navarro et al. 2017). Likewise, (–)‐epigallocatechin has been reported to activate FOXO3a, a transcription factor that regulates antioxidant enzymes, including superoxide dismutase and catalase (Bartholome et al. 2010). In addition, poor sleep quality is linked to reduced respiratory center sensitivity to chemical, mechanical, and cortical signals, especially during REM sleep (DeMarco Jr. et al. 1981; Mulloy and McNicholas 1996). This disruption in ventilation‐perfusion matching may lead to hypoxemia, aggravating the nocturnal oxygen desaturation caused by the normal hypoventilation that occurs during sleep (McNicholas 2000). These observations suggest that inadequate dietary antioxidant intake and poor sleep quality may independently produce harmful biological effects, potentially explaining their combined influence on COPD risk.

However, cross‐sectional studies can only identify associations rather than prove causality. To address this limitation, we conducted a MR analysis to determine whether diet and sleep have causal effects on COPD. In this analysis, the IVW method showed that genetic variants associated with diet and sleep may collectively increase COPD risk. These findings remained stable across sensitivity analyses, implying that diet and sleep may contribute to COPD onset. A recent MR analysis supported a causal link between genetically predicted dietary intake and COPD (Zhang et al. 2024), consistent with our findings. To our knowledge, our MR analysis represents an early attempt to investigate the causal link between sleep quality and COPD, providing initial evidence that sleep quality may be a causal factor for the disease.

The limitations of this study involve its observational and genetic components. The generalizability of the NHANES‐based observational findings is limited by its US focus and the potential for recall bias in self‐reported data. For the MR analysis, key limitations include the European ancestry bias in the source GWAS and potential sample overlap, which despite sensitivity analyses, may affect the robustness of the causal inferences.

## 5 Conclusions

Using a nationally representative dataset and MR analysis, our results demonstrated significant relationships linking overall dietary antioxidant intake and sleep quality with COPD. Furthermore, this study is the first to identify a combined protective effect of high dietary antioxidant intake and adequate sleep quality against the risk of developing COPD. These observations underscore the importance of integrating sleep quality and overall dietary antioxidant intake when developing targeted interventions for COPD prevention.

## Author Contributions

**Kaiyue Wang:** data curation (equal), formal analysis (equal), investigation (equal), visualization (equal), writing – original draft (equal), writing – review and editing (equal). **Xinjing Lou:** data curation (equal), formal analysis (equal), investigation (equal), visualization (equal), writing – original draft (equal), writing – review and editing (equal). **Shenghan Wang:** data curation (equal), investigation (equal), validation (equal). **Qunhao Huang:** investigation (equal). **Lingna Liu:** investigation (equal). **Jiangnan Lin:** investigation (equal). **Linyu Wu:** conceptualization (equal), data curation (equal), investigation (equal), methodology (equal), project administration (equal), resources (equal), supervision (equal), writing – review and editing (equal). **Chen Gao:** conceptualization (equal), data curation (equal), investigation (equal), methodology (equal), project administration (equal), resources (equal), supervision (equal), writing – review and editing (equal).

## Ethics Statement

As a publicly available National Center for Health Statistics (NCHS) resource, data from the National Health and Nutrition Examination Survey (NHANES) were acquired with full IRB oversight and written participant consent. This study's retrospective analysis of de‐identified records qualifies for exemption per US Department of Health and Human Services policy (Category 4 exemption), requiring no new ethical approval.

## Conflicts of Interest

The authors declare no conflicts of interest.

## Supporting information


**Figure S1:** Subgroup analysis of DAQS and COPD. Adjusted for age, sex, race, body mass index (BMI), smoking status, alcohol consumption, history of hypertension, history of diabetes, and systemic immune‐inflammation index (SII).

**Figure S2:** Subgroup analysis of PSQI and COPD. Adjusted for age, sex, race, body mass index (BMI), smoking status, alcohol consumption, history of hypertension, history of diabetes, and systemic immune‐inflammation index (SII).

**Figure S3:** Subgroup analysis of the combined effect of DAQS and PSQI with COPD. Adjusted for age, sex, race, body mass index (BMI), smoking status, alcohol consumption, history of hypertension, history of diabetes, and systemic immune‐inflammation index (SII).

**Figure S4:** Diet and Sleep‐COPD two‐sample MR analysis and sensitivity analysis. Forest plot showing results from the two‐sample Mendelian randomization study to assess associations between variation in diet, sleep duration and chronic obstructive pulmonary disease (COPD). CI, confidence interval; OR, odds ratio.

**Table S1:** Subgroups with significant combined effects.

**Table S2:** The F‐statistic of Mendelian randomization for sleep duration.

**Table S3:** The F‐statistic of Mendelian randomization for variation in diet.

**Table S4:** The F‐statistic of multivariate Mendelian randomization.

## Data Availability

The NHANES dataset utilized in this investigation is publicly accessible through the National Center for Health Statistics repository (https://wwwn.cdc.gov/nchs/nhanes), adhering to CDC's data sharing policies for secondary research.
