# Supplementary figures and images for: Dietary Antioxidant Intake and Sleep Quality: Combined Effects on Chronic Obstructive Pulmonary Disease in NHANES 2005–2008 and Mendelian Randomization Analysis
Source: Food Sci Nutr. 2025 Nov 17;13(11):e71209. doi: 10.1002/fsn3.71209 (PMC12620672; doi:10.1002/fsn3.71209)

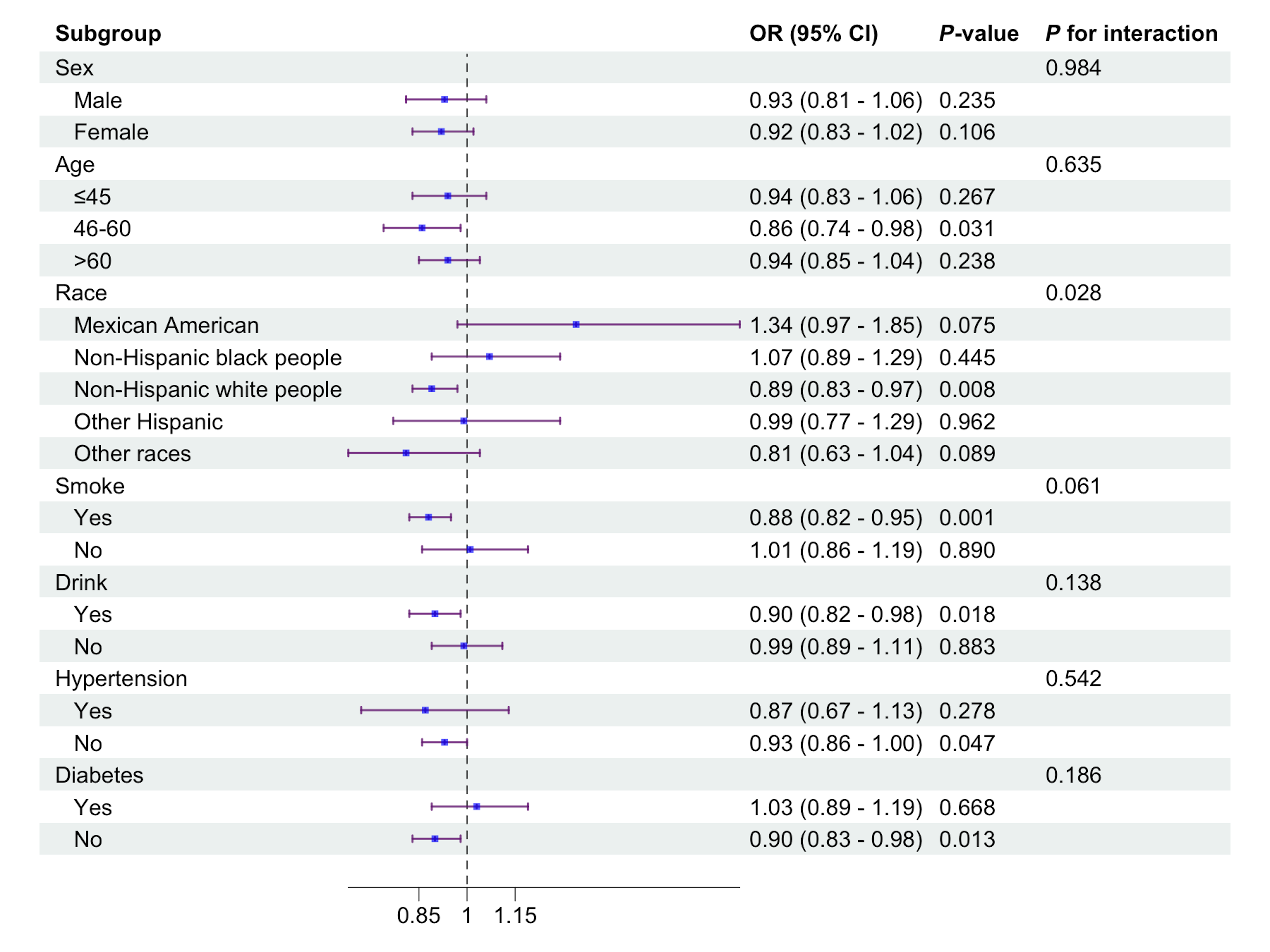

Supplement: Supplementary file 1 — Figure S1: Subgroup analysis of DAQS and COPD. Adjusted for age, sex, race, body mass index (BMI), smoking status, alcohol consumption, history of hypertension, history of diabetes, and systemic immune‐inflammation index (SII). [file FSN3-13-e71209-s005.tiff]

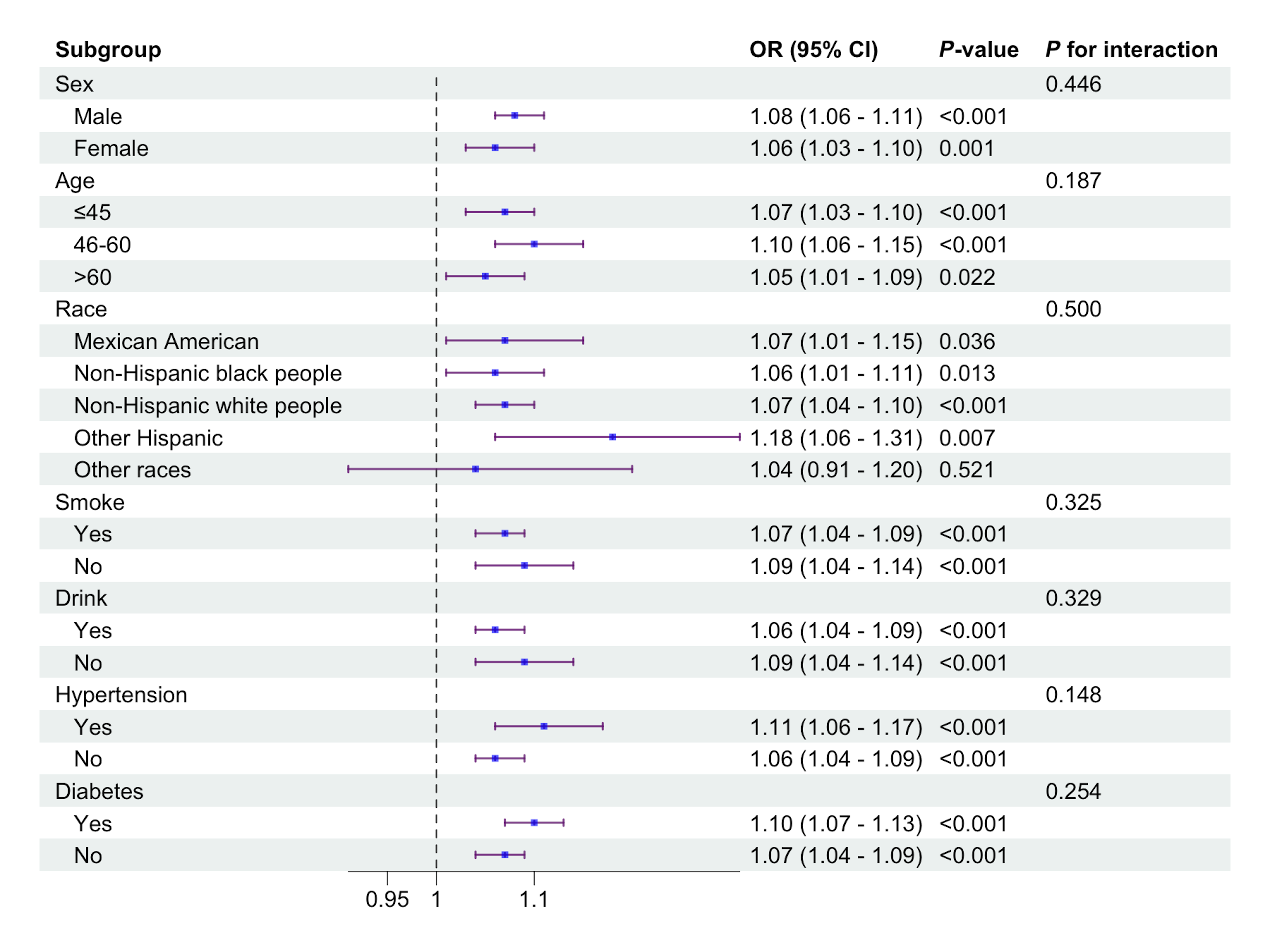

Supplement: Supplementary file 2 — Figure S2: Subgroup analysis of PSQI and COPD. Adjusted for age, sex, race, body mass index (BMI), smoking status, alcohol consumption, history of hypertension, history of diabetes, and systemic immune‐inflammation index (SII). [file FSN3-13-e71209-s007.tiff]

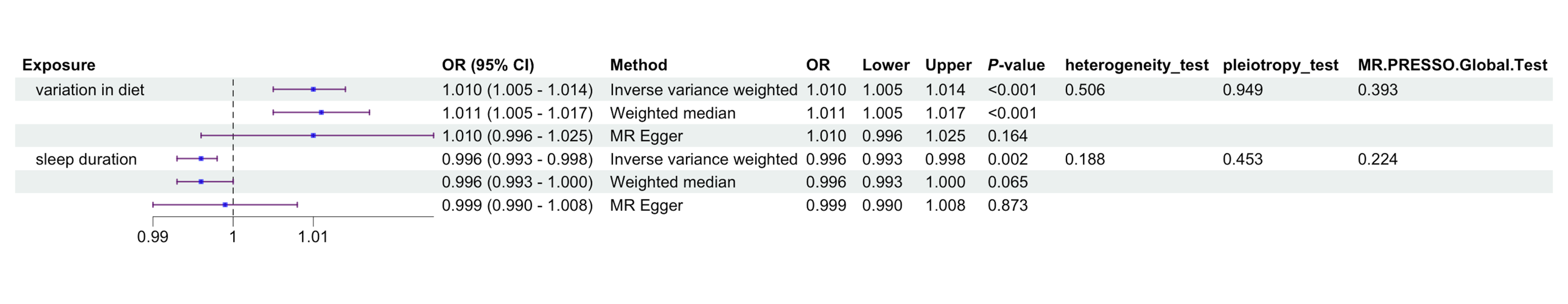

Supplement: Supplementary file 4 — Figure S4: Diet and Sleep‐COPD two‐sample MR analysis and sensitivity analysis. Forest plot showing results from the two‐sample Mendelian randomization study to assess associations between variation in diet, sleep duration and chronic obstructive pulmonary disease (COPD). CI, confidence interval; OR, odds ratio. [file FSN3-13-e71209-s004.tiff]
