# Supplementary material for: Dietary Antioxidant Intake and Sleep Quality: Combined Effects on Chronic Obstructive Pulmonary Disease in NHANES 2005–2008 and Mendelian Randomization Analysis
Source: Food Sci Nutr. 2025 Nov 17;13(11):e71209. doi: 10.1002/fsn3.71209 (PMC12620672; doi:10.1002/fsn3.71209)
Supplement: Supplementary file 5 — Table S1: Subgroups with significant combined effects. [file FSN3-13-e71209-s001.docx]

Table S1 Subgroups with significant combined effects

| Subgroups | RERI | ROR |
| --- | --- | --- |
| **Gender** |  |  |
| Male | 0.30 | 1.35 |
| Female | 0.12 | 1.06 |
| **Age** |  |  |
| 46-60 | 0.43 | 1.65 |
| >60 | 0.21 | 1.16 |
| **Race** |  |  |
| Non-Hispanic white People | 0.19 | 1.10 |
| **Smoke** |  |  |
| Yes | 0.29 | 1.33 |
| **Drink** |  |  |
| Yes | 0.16 | 1.07 |
| **Diabetes** |  |  |
| No | 0.11 | 1.02 |
