# Supplementary material for: Dietary Antioxidant Intake and Sleep Quality: Combined Effects on Chronic Obstructive Pulmonary Disease in NHANES 2005–2008 and Mendelian Randomization Analysis
Source: Food Sci Nutr. 2025 Nov 17;13(11):e71209. doi: 10.1002/fsn3.71209 (PMC12620672; doi:10.1002/fsn3.71209)
Supplement: Supplementary file 6 — Table S2: The F‐statistic of Mendelian randomization for sleep duration. [file FSN3-13-e71209-s002.docx]

Table S2 The F-statistic of Mendelian randomization for sleep duration

| SNP | F-statistic |
| --- | --- |
| rs2863957 | 224.459174 |
| rs2683630 | 81.5565553 |
| rs75539574 | 67.789634 |
| rs13107325 | 63.7673184 |
| rs34556183 | 56.1807698 |
| rs9302680 | 55.9117577 |
| rs8038326 | 55.7115204 |
| rs2079070 | 55.0518223 |
| rs915416 | 52.276668 |
| rs1517572 | 51.6784942 |
| rs56367859 | 50.4730836 |
| rs1348047 | 48.2230835 |
| rs6889592 | 48.0698691 |
| rs12567114 | 47.2824378 |
| rs8047587 | 46.6842342 |
| rs11621908 | 46.1050076 |
| rs113021516 | 45.7557127 |
| rs7115856 | 45.5639108 |
| rs62444917 | 45.290367 |
| rs34786000 | 45.3097271 |
| rs2186122 | 44.6847314 |
| rs4767550 | 44.3541119 |
| rs11650677 | 43.7049772 |
| rs7831557 | 43.5327198 |
| rs11039216 | 41.0616502 |
| rs1972712 | 40.7627075 |
| rs11982852 | 39.6364109 |
| rs1939455 | 39.3931503 |
| rs7644809 | 39.0438445 |
| rs12518468 | 39.0661703 |
| rs11643715 | 38.6878559 |
| rs2192528 | 37.5026317 |
| rs7517981 | 37.1977636 |
| rs6783516 | 36.3750614 |
| rs35662245 | 36.0880621 |
| rs2734831 | 35.7517188 |
| rs374153 | 35.5477889 |
| rs7016314 | 35.0935391 |
| rs9611007 | 35.0171145 |
| rs9903898 | 34.8257308 |
| rs76258078 | 34.7053191 |
| rs9810474 | 34.6859081 |
| rs6561715 | 34.6853628 |
| rs2839753 | 34.4416308 |
| rs17391944 | 34.4312266 |
| rs174564 | 33.7295136 |
| rs55658675 | 33.5138134 |
| rs10510128 | 33.3591653 |
| rs365663 | 33.2458919 |
| rs1553132 | 33.2532018 |
| rs113113059 | 33.1292143 |
| rs9382445 | 33.0766062 |
| rs6681755 | 33.0542698 |
| rs2072727 | 32.9805586 |
| rs151014368 | 32.7574322 |
| rs8074498 | 32.5947825 |
| rs7711696 | 32.3313109 |
| rs34354917 | 32.1208763 |
| rs72771082 | 32.1345139 |
| rs9345234 | 32.0574515 |
| rs2748809 | 31.6242101 |
| rs35126035 | 31.2942972 |
| rs1463053 | 31.1322259 |
| rs112100783 | 30.9258954 |
| rs2236295 | 30.7619255 |
| rs2279681 | 30.4619473 |
| rs72831782 | 30.4899572 |
| rs7740402 | 30.0903053 |
| rs8072993 | 30.0491581 |
| rs17732997 | 29.8577886 |
| rs56337305 | 29.6397694 |
| rs12501164 | 29.6518724 |
| rs41848 | 29.4112258 |
| rs2298432 | 28.9960689 |
| rs3027234 | 28.8510285 |
| rs11043328 | 28.7467591 |
| rs2249966 | 28.1645118 |
| rs4364707 | 27.9671233 |
| rs12791153 | 27.9057409 |
| rs61937595 | 27.8889509 |
| rs12287601 | 27.7011736 |
| rs13390171 | 27.6183991 |
| rs11185890 | 27.5990873 |
| rs12321904 | 27.5385106 |
| rs16840048 | 27.5098516 |
| rs146390523 | 27.4192831 |
| rs2161853 | 27.3830143 |
| rs13112591 | 27.3801534 |
| rs11738968 | 27.3722276 |
| rs4588900 | 27.3021809 |
| rs62043048 | 27.3191567 |
| rs17073339 | 27.2100996 |
| rs12256551 | 27.2215179 |
| rs73208119 | 27.2264153 |
| rs3957781 | 27.124977 |
| rs17767831 | 27.1428247 |
| rs2231265 | 27.1240942 |
| rs61985058 | 27.1633929 |
| rs79783687 | 27.0961062 |
| rs6092702 | 27.1567099 |
| rs12760654 | 27.0233788 |
| rs58153210 | 27.0138213 |
| rs72863018 | 26.9042341 |
| rs55938136 | 26.9634552 |
| rs4659554 | 26.87109 |
| rs7132277 | 26.7600285 |
| rs4428289 | 26.5237486 |
| rs9842017 | 26.460586 |
| rs12598706 | 26.4746766 |
| rs12670234 | 26.2726758 |
| rs62180559 | 26.1773651 |
| rs138038141 | 26.078576 |
| rs6470491 | 26.0383272 |
| rs8027897 | 26.0270661 |
| rs4688116 | 25.9715271 |
| rs61813324 | 25.9204198 |
| rs67649350 | 25.9137869 |
| rs61783168 | 25.861956 |
| rs1245192 | 25.7384673 |
| rs1246339 | 25.6799674 |
| rs76687186 | 25.662778 |
| rs7633013 | 25.4962739 |
| rs11151115 | 25.3680431 |
| rs2588692 | 25.2947672 |
| rs12336359 | 25.2167013 |
| rs799834 | 25.1335515 |
| rs258700 | 25.138596 |
| rs80071665 | 25.0342572 |
| rs11135570 | 25.0279352 |
| rs269054 | 24.9751979 |
| rs62120041 | 24.9571941 |
| rs6932158 | 24.9505987 |
| rs74752557 | 24.8896582 |
| rs1323342 | 24.8259026 |
| rs62493145 | 24.65092 |
| rs10863936 | 24.6097931 |
| rs11190970 | 24.5456996 |
| rs4924071 | 24.4417762 |
| rs10873481 | 24.4060926 |
| rs35758221 | 24.3620725 |
| rs35788479 | 24.3139066 |
| rs16869109 | 24.2913232 |
| rs67988891 | 24.1561233 |
| rs62009949 | 24.1133688 |
| rs13066140 | 24.0665785 |
| rs10770596 | 23.9665538 |
| rs35267052 | 23.891402 |
| rs4246177 | 23.9197438 |
| rs28828150 | 23.7193921 |
| rs11222509 | 23.8155382 |
| rs4887173 | 23.8302562 |
| rs72825773 | 23.7780454 |
| rs113817466 | 23.6636289 |
| rs77400460 | 23.5494515 |
| rs138226610 | 23.5590413 |
| rs13197257 | 23.4065618 |
| rs4559781 | 23.4723716 |
| rs4777272 | 23.4450781 |
| rs4790581 | 23.3540452 |
| rs4014158 | 23.2536557 |
| rs151238700 | 23.261333 |
| rs73194727 | 23.2509966 |
| rs72676659 | 23.2422249 |
| rs10173260 | 23.1910266 |
| rs1019306 | 22.9808997 |
| rs1019046 | 23.0294136 |
| rs17778974 | 22.9706483 |
| rs7250230 | 22.9859129 |
| rs72784352 | 22.9192138 |
| rs6492059 | 22.8777905 |
| rs221420 | 22.8607207 |
| rs10911599 | 22.7526845 |
| rs11779521 | 22.8449869 |
| rs7951019 | 22.7999753 |
| rs2497765 | 22.6052076 |
| rs4489806 | 22.6164195 |
| rs7598252 | 22.5219385 |
| rs76295369 | 22.540197 |
| rs803728 | 22.5105943 |
| rs8045843 | 22.5319663 |
| rs73166891 | 22.5347303 |
| rs34850165 | 22.3706373 |
| rs4815270 | 22.3893457 |
| rs8103135 | 22.2334007 |
| rs1858588 | 22.2042714 |
| rs114052004 | 22.1600032 |
| rs55895313 | 22.1855581 |
| rs10820727 | 22.131302 |
| rs61818679 | 22.0715687 |
| rs116610008 | 22.120869 |
| rs401966 | 22.0735222 |
| rs1819187 | 22.023879 |
| rs4491389 | 21.9570369 |
| rs73415247 | 21.8972057 |
| rs1526770 | 21.8190968 |
| rs56072518 | 21.836091 |
| rs10788612 | 21.8471282 |
| rs112346612 | 21.8260039 |
| rs78677597 | 21.7381459 |
| rs7464481 | 21.6844571 |
| rs73497985 | 21.6224847 |
| rs12001843 | 21.5521362 |
| rs9804910 | 21.5781636 |
| rs177912 | 21.4421793 |
| rs1994651 | 21.4872059 |
| rs17146792 | 21.492983 |
| rs6986416 | 21.4015729 |
| rs148251429 | 21.3050574 |
| rs7943526 | 21.2509322 |
| rs17725296 | 21.2177594 |
| rs7037083 | 21.2410163 |
| rs1008078 | 21.1767867 |
| rs10927132 | 21.0978595 |
| rs4885746 | 21.0985519 |
| rs16900563 | 21.029194 |
| rs6481433 | 21.0535746 |
| rs10899421 | 21.0476878 |
| rs8015629 | 21.026477 |
| rs12431506 | 20.9799678 |
| rs3958355 | 20.9731387 |
| rs664969 | 20.9745727 |
| rs79045904 | 20.9398111 |
| rs6083860 | 20.9568253 |
| rs75636080 | 20.8968802 |
| rs117321464 | 20.8889316 |
| rs11016146 | 20.8034251 |
| rs9371028 | 20.7254271 |
| rs28652618 | 20.690686 |
| rs1016680 | 20.6820662 |
| rs28374712 | 20.6381357 |
| rs9643333 | 20.670287 |
| rs6043178 | 20.6585606 |
| rs921169 | 20.6141503 |
| rs2059877 | 20.5938163 |
| rs7794915 | 20.4899619 |
| rs10978476 | 20.4723585 |
| rs588232 | 20.4456723 |
| rs117914880 | 20.3962985 |
| rs56129102 | 20.3144655 |
| rs4559549 | 20.2454557 |
| rs6829198 | 20.2180045 |
| rs3801290 | 20.1894758 |
| rs61811629 | 20.1766015 |
| rs2373289 | 20.1081018 |
| rs35938338 | 20.0512292 |
| rs34352619 | 20.0265705 |
| rs464930 | 20.0115848 |
| rs10061466 | 19.9789742 |
| rs1871900 | 19.9759866 |
| rs113177297 | 19.8652103 |
| rs4692256 | 19.8468788 |
| rs73254474 | 19.8262173 |
| rs17666399 | 19.7791757 |
| rs72739772 | 19.786076 |
| rs35589090 | 19.7805327 |
| rs1483007 | 19.6890702 |
| rs2499617 | 19.6363185 |
| rs59226301 | 19.6055582 |
| rs73187206 | 19.550036 |
| rs13013224 | 19.528638 |
| rs9577206 | 19.5396552 |
| rs9901223 | 19.5273053 |
