# Supplementary material for: Dietary Antioxidant Intake and Sleep Quality: Combined Effects on Chronic Obstructive Pulmonary Disease in NHANES 2005–2008 and Mendelian Randomization Analysis
Source: Food Sci Nutr. 2025 Nov 17;13(11):e71209. doi: 10.1002/fsn3.71209 (PMC12620672; doi:10.1002/fsn3.71209)
Supplement: Supplementary file 7 — Table S3: The F‐statistic of Mendelian randomization for variation in diet. [file FSN3-13-e71209-s006.docx]

Table S3 The F-statistic of Mendelian randomization for variation in diet

| SNP | F-statistic |
| --- | --- |
| rs7634084 | 53.1942312 |
| rs1421085 | 50.4715624 |
| rs997467 | 48.6870824 |
| rs606725 | 47.5917813 |
| rs728083 | 46.1987309 |
| rs28637922 | 41.8136184 |
| rs4548895 | 34.8540436 |
| rs1438900 | 33.2130604 |
| rs11749676 | 33.2005328 |
| rs1517572 | 31.6710862 |
| rs10133551 | 31.746062 |
| rs1561419 | 31.6301542 |
| rs12148513 | 31.2597356 |
| rs9616911 | 31.0548341 |
| rs6063438 | 30.9006049 |
| rs7027515 | 30.2761172 |
| rs6057936 | 28.9762861 |
| rs2643434 | 28.7037067 |
| rs12079233 | 28.6154567 |
| rs7724146 | 28.355146 |
| rs2020235 | 28.1310312 |
| rs2126336 | 28.1118735 |
| rs1919584 | 28.0815713 |
| rs2798282 | 27.8336053 |
| rs838133 | 27.8232247 |
| rs2722010 | 27.4428825 |
| rs6483830 | 27.4242153 |
| rs598330 | 27.4657767 |
| rs324017 | 27.1751908 |
| rs1862450 | 27.1544362 |
| rs1894588 | 27.1053365 |
| rs35284121 | 27.0310379 |
| rs6668309 | 26.8961336 |
| rs2654808 | 26.8287807 |
| rs72821273 | 26.8197286 |
| rs11779468 | 26.87937 |
| rs58024485 | 26.8007249 |
| rs7243387 | 26.3195884 |
| rs11207177 | 26.2583211 |
| rs74709145 | 26.1945761 |
| rs28478765 | 26.168598 |
| rs17565470 | 26.1830484 |
| rs10157166 | 26.0353723 |
| rs71578225 | 25.8691064 |
| rs1190234 | 25.7585163 |
| rs7958081 | 25.6839091 |
| rs10791889 | 25.5598921 |
| rs7900590 | 25.5004708 |
| rs2394102 | 25.4376654 |
| rs146247102 | 25.37516 |
| rs2633793 | 25.341303 |
| rs7572865 | 25.3139583 |
| rs17199009 | 25.2810315 |
| rs117666098 | 25.22302 |
| rs4805950 | 25.1767372 |
| rs79764489 | 25.1049376 |
| rs4628086 | 24.8486201 |
| rs112654635 | 24.7862008 |
| rs750472 | 24.7366995 |
| rs17712032 | 24.3236493 |
| rs4027581 | 24.0307072 |
| rs1338123 | 23.9597033 |
| rs6426837 | 23.6886616 |
| rs7620948 | 23.6842798 |
| rs7300633 | 23.3862141 |
| rs61770510 | 23.2642596 |
| rs1567421 | 23.2533943 |
| rs4423760 | 23.3175516 |
| rs16853589 | 23.2015034 |
| rs74824115 | 22.8658454 |
| rs12663341 | 22.880809 |
| rs11669756 | 22.9525083 |
| rs2976573 | 22.7423648 |
| rs4664747 | 22.5844144 |
| rs183178911 | 22.5833587 |
| rs72777303 | 22.6365488 |
| rs4701928 | 22.5216752 |
| rs72783895 | 22.5130107 |
| rs1889986 | 22.5331939 |
| rs702130 | 22.510006 |
| rs7539088 | 22.3018141 |
| rs62182813 | 22.3567853 |
| rs12527158 | 22.2946662 |
| rs62003971 | 22.2912688 |
| rs11608693 | 22.2281371 |
| rs41488953 | 22.1615006 |
| rs6676159 | 22.048814 |
| rs10173724 | 21.8899594 |
| rs3770774 | 21.8115522 |
| rs6943379 | 21.7949483 |
| rs7229677 | 21.7994168 |
| rs2468314 | 21.7304664 |
| rs62155398 | 21.6985711 |
| rs150197496 | 21.6607798 |
| rs7074375 | 21.6523281 |
| rs72632745 | 21.5145987 |
| rs147203243 | 21.4512743 |
| rs77468362 | 21.4145931 |
| rs4671212 | 21.2969687 |
| rs13006224 | 21.3249124 |
| rs613683 | 21.3219715 |
| rs7117234 | 21.2752008 |
| rs4572145 | 21.2824323 |
| rs1744291 | 21.201085 |
| rs10439469 | 21.1628904 |
| rs77315417 | 21.1872033 |
| rs10465566 | 21.1288566 |
| rs1381138 | 21.1448578 |
| rs9675315 | 21.0714989 |
| rs17210284 | 21.0535136 |
| rs7634114 | 20.9694381 |
| rs10852404 | 20.8607952 |
| rs115711980 | 20.8349368 |
| rs11125302 | 20.8517509 |
| rs78901372 | 20.7065199 |
| rs471791 | 20.6216583 |
| rs9919303 | 20.582809 |
| rs10984352 | 20.6018554 |
| rs3099950 | 20.538777 |
| rs13076741 | 20.5567149 |
| rs1552619 | 20.5564678 |
| rs2289447 | 20.4600403 |
| rs35102004 | 20.4167032 |
| rs12904524 | 20.4195834 |
| rs6593941 | 20.3915017 |
| rs2674032 | 20.3848243 |
| rs113029978 | 20.4024506 |
| rs138080759 | 20.3938178 |
| rs16913576 | 20.3209399 |
| rs7748725 | 20.2847971 |
| rs4771744 | 20.2114981 |
| rs6841167 | 20.1944334 |
| rs143321361 | 20.144842 |
| rs4820988 | 20.1302233 |
| rs1647778 | 20.1044387 |
| rs12043534 | 20.0965728 |
| rs72700567 | 20.0547241 |
| rs11146213 | 20.0554954 |
| rs7148293 | 20.0185438 |
| rs111386387 | 19.9999123 |
| rs35305377 | 19.965181 |
| rs12785722 | 19.9639888 |
| rs61735591 | 19.9345815 |
| rs9309664 | 19.915145 |
| rs7583765 | 19.8676077 |
| rs76412127 | 19.8687875 |
| rs114752106 | 19.8552915 |
| rs147513171 | 19.8276761 |
| rs865342 | 19.810326 |
| rs2250230 | 19.7999925 |
| rs1256430 | 19.7949822 |
| rs137875467 | 19.794269 |
| rs10858271 | 19.7388013 |
| rs12792273 | 19.7190833 |
| rs35846993 | 19.7018989 |
| rs146592304 | 19.6650486 |
| rs1054428 | 19.6571583 |
| rs73140000 | 19.5626919 |
