# Supplementary material for: Dietary Antioxidant Intake and Sleep Quality: Combined Effects on Chronic Obstructive Pulmonary Disease in NHANES 2005–2008 and Mendelian Randomization Analysis
Source: Food Sci Nutr. 2025 Nov 17;13(11):e71209. doi: 10.1002/fsn3.71209 (PMC12620672; doi:10.1002/fsn3.71209)
Supplement: Supplementary file 8 — Table S4: The F‐statistic of multivariate Mendelian randomization. [file FSN3-13-e71209-s003.docx]

Table S4 The F-statistic of multivariate Mendelian randomization

|  | sleep duration | variation in diet |
| --- | --- | --- |
| F-statistic | 8.372243 | 21.42533 |
